# A Typical Dermoscopic Pattern of PLEVA

**DOI:** 10.1002/ccr3.70686

**Published:** 2025-07-27

**Authors:** Mahesh Mathur, Sumit Paudel, Supriya Paudel, Sambidha Karki, Sandhya Regmi, Nabita Bhattarai

**Affiliations:** ^1^ Department of Dermatology, College of Medical Sciences Teaching Hospital Bharatpur Nepal

**Keywords:** central crust, dermoscopy, PLEVA, targetoid pattern, vascular structures, white scales

## Abstract

PLEVA is a benign inflammatory disorder. Dermoscopy is a non‐invasive diagnostic modality. Thus, the new dermoscopic pattern mentioned in our literature can aid in the diagnosis of the disease, unlike the skin biopsy, which is an invasive modality and shows non‐specific features in PLEVA.

**Abbreviations**
PLEVA: pityriasis licenoides et varioliformis acute
RBCs: Red blood cells

**Summary**

- What is already known about this topic?
  ○ PLEVA is characterized by erythematous papules, vesicles, pustules, ulcers, and mica‐like scale that heals with varicella‐like scars. Dermoscopic features mentioned in the literature are white‐colored structureless areas, a central crust, red globules, blue‐gray areas, and scalings.
- What does this study add?
  ○ Our case report presents slightly different dermoscopic features of PLEVA. We observed a typical target pattern of central crust, middle vascular zone, and peripheral whitish scales, which has not been reported in literature so far. This new dermoscopic pattern, as described in our case, can aid in the diagnosis of PLEVA.

## 1 Introduction

Pityriasis lichenoides et varioliformis acuta (PLEVA) is a rare cutaneous inflammatory condition affecting children and young adults. It is characterized by erythematous papules, vesicles, pustules, ulcers, and mica‐like scales that heal with varicella‐like scars. PLEVA mainly involves the trunk and proximal extremities and has a slight male predominance [1].

The etiology includes an inflammatory reaction triggered by bacterial, viral, or protozoal infection, immunizations, an immune response secondary to T‐cell dyscrasia, and an immune complex‐mediated hypersensitivity reaction [2].

Histopathology of skin lesions is nonspecific. Diagnosis is mainly clinical, aided by dermoscopy [1]. Dermoscopic patterns in PLEVA include white‐colored structureless areas, a central crust, red globules, blue‐gray areas, and scalings [3]. Hereby, we present a case of PLEVA with newer dermoscopic findings.

## 2 Case Presentation

**FIGURE 1:**
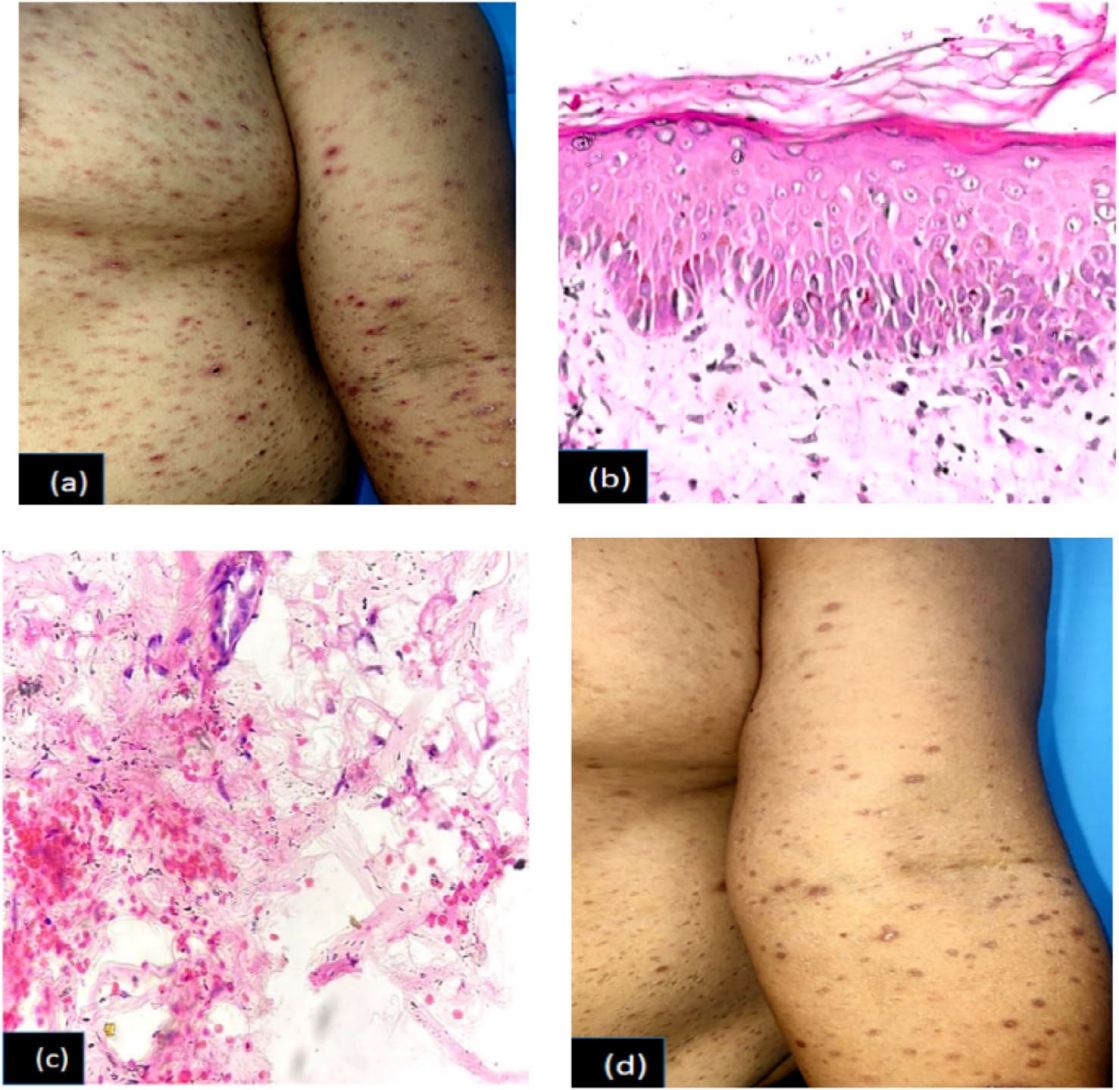
(a) PLEVA: Mulitple erythematous macules, papules, crusted lesions with mica like scales over trunk and extremities, 1 (b) Hematoxylin and eosin (H/E) stain (40×): Parakeratosis, spongiosis and vacuolar degeneration in basal layer of epidermis, 1 (c) H/E stain (40×): Extravasation of RBCs in dermis, 1 (d) Multiple hyperpigmented macules and papules following 3 weeks of treatment with doxycycline.

A 41‐year‐old male presented with multiple, asymptomatic, erythematous, crusted papules and hyperpigmented macules over bilateral upper and lower extremities, anterior and posterior trunk, sparing face, palms, and soles for a 1‐month duration (Figure 1a). Routine investigations like complete blood counts and serology were done. Lab reports were within normal limits. There was no history of recent vaccination.

## 3 Methods

**FIGURE 2:**
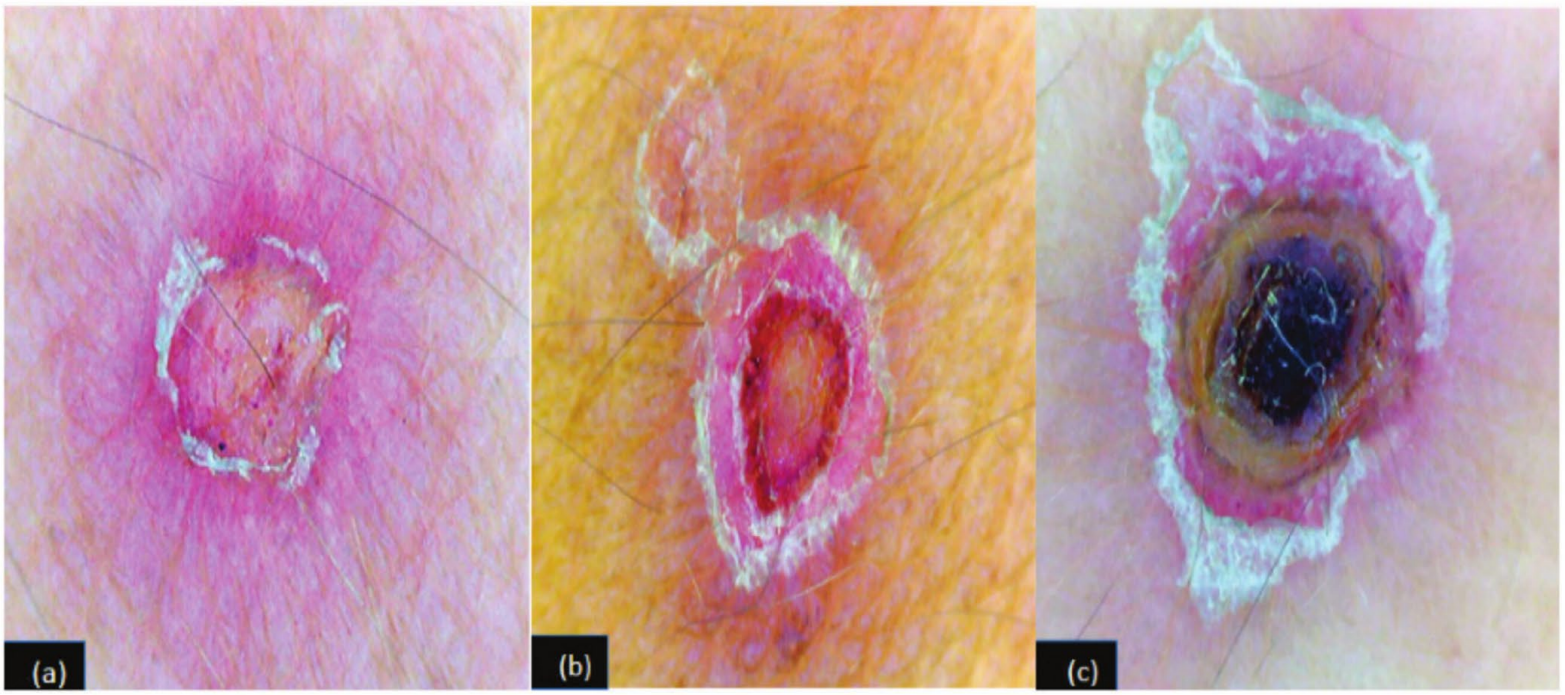
(a) Dermoscopy PLEVA: Central crusting, reddish areas with polymorphic vessels surrounded by peripheral white scales, 2 (b) Typical target pattern of central crust, intermediate vascular ring surrounded by white scales on pinkish background, 2 (c) Central necrotic areas around the hair follicles with peripheral crusting and scaling.

Dermoscopy of a few lesions showed central crustings, reddish areas with dotted and linear vessels surrounded by peripheral white scales (Figure 2a), while the majority of lesions had a target pattern of central crust, an intermediate vascular ring surrounded by white scales on a pinkish background (Figure 2b). Some lesions also revealed central necrotic areas around the hair follicles with peripheral crustings and scalings (Figure 2c).

Histopathological examination of the lesion from the left forearm demonstrated focal parakeratosis, spongiosis, and vacuolar degeneration in the basal layer with lymphocytic and neutrophilic infiltrations, epidermal exocytosis, and extravasation of RBCs (Figure 1b,c).

## 4 Results

On the basis of clinical, dermoscopic, and histopathology reports, a diagnosis of PLEVA was made. The patient was counseled and prescribed doxycycline for 3 weeks, with significant improvement noted in the 3‐week follow‐up (Figure 1d).

## 5 Discussion

PLEVA is an acute polymorphic eruption of erythematous macules quickly evolving into papules with a fine micaceous scale eventually progressing to vesicles, pustules, hemorrhagic and necrotic crusts lasting from weeks to months. Lesions heal with varioliform scars, postinflammatory hyperpigmentation, and hypopigmentation [2].

The histopathology of PLEVA is characterized by spongiosis, parakeratosis, acanthosis, intraepidermal vesicles, necrosis, wedge‐shaped dermal lymphohistiocytic inflammatory infiltrate, and erythrocyte extravasations, as observed in our case.

Dermoscopy and histopathological correlation were described by Ankad and Beergouder, in which an amorphous brownish structure corresponds to basophilic material in the epidermis and wedge‐shaped lymphocytic infiltration in the dermis. Red dots and hemorrhages represent extravasations of red blood cells in the papillary dermis and dilatation of blood vessels. Whitish, structureless, and crusted areas are due to hyperkeratosis, acanthosis, and epidermal erosion [3].

Dermoscopic features as mentioned in literature consist of three concentric zones of central brownish clod, intermediate ring of white scale, and peripheral vascular ring [3]. However, in our case, in the majority of lesions, we observed a typical target pattern of central crust, middle vascular zone, and peripheral whitish scales, which has not been reported in the literature so far.

The differential diagnosis for PLEVA includes lymphomatoid papulosis, arthropod bite reactions, varicella, Gianotti‐Crosti syndrome, erythema multiforme, pityriasis rosea, guttate psoriasis, and secondary syphilis [2]. Various treatment modalities include doxycycline, erythromycin, acyclovir, dapsone, methotrexate, psoralen plus ultraviolet A, infliximab, and etanercept. Systemic corticosteroids have a role in severe cases of PLEVA [4].

## 6 Conclusion

PLEVA mimics many other cutaneous conditions, and histopathology is not pathognomonic. Thus, the new dermoscopic pattern as described in our case can aid in the diagnosis of PLEVA.

## Author Contributions

**Mahesh Mathur:** conceptualization, data curation, supervision, validation. **Sumit Paudel:** conceptualization, data curation, supervision, validation. **Supriya Paudel:** formal analysis, methodology, resources, visualization. **Sambidha Karki:** conceptualization, project administration, resources, visualization. **Sandhya Regmi:** investigation, software, validation, visualization. **Nabita Bhattarai:** conceptualization, data curation, supervision, validation.

## Ethics Statement

Reviewed and approved by Institutional Review Board College of Medical Sciences (IRBCOMS). The patients in this manuscript have given written informed consent to the publication of their case details.

## Consent

The authors obtained written consent from the patient for the use of photographs and medical information to be published online and with the understanding that this information may be publicly available and discoverable via search engines. Patient consent forms are not provided to the journal but are retained by the authors.

## Conflicts of Interest

The authors declare no conflicts of interest.

## Data Availability

The data that support the findings of this study are available from the corresponding author upon reasonable request.
